# Targeting Ferroptosis as a Promising Therapeutic Strategy for Ischemia-Reperfusion Injury

**DOI:** 10.3390/antiox11112196

**Published:** 2022-11-06

**Authors:** Yihang Pan, Xueke Wang, Xiwang Liu, Lihua Shen, Qixing Chen, Qiang Shu

**Affiliations:** 1 Department of Clinical Research Center, The Children’s Hospital, School of Medicine, Zhejiang University, National Clinical Research Center for Child Health, Hangzhou 310052, China; 2 Department of Thoracic & Cardiovascular Surgery, The Children’s Hospital, School of Medicine, Zhejiang University, Hangzhou 310003, China; 3 Key Laboratory of Diagnosis and Treatment of Neonatal Diseases of Zhejiang Province, Hangzhou 310052, China

**Keywords:** ischemia-reperfusion injury, ferroptosis, iron, antioxidant, therapeutic strategies

## Abstract

Ischemia-reperfusion (I/R) injury is a major challenge in perioperative medicine that contributes to pathological damage in various conditions, including ischemic stroke, myocardial infarction, acute lung injury, liver transplantation, acute kidney injury and hemorrhagic shock. I/R damage is often irreversible, and current treatments for I/R injury are limited. Ferroptosis, a type of regulated cell death characterized by the iron-dependent accumulation of lipid hydroperoxides, has been implicated in multiple diseases, including I/R injury. Emerging evidence suggests that ferroptosis can serve as a therapeutic target to alleviate I/R injury, and pharmacological strategies targeting ferroptosis have been developed in I/R models. Here, we systematically summarize recent advances in research on ferroptosis in I/R injury and provide a comprehensive analysis of ferroptosis-regulated genes investigated in the context of I/R, as well as the therapeutic applications of ferroptosis regulators, to provide insights into developing therapeutic strategies for this devastating disease.

## 1. Introduction

Ischemia-reperfusion (I/R) is a pathological condition characterized by the initial restriction of the blood supply to organs or tissues, followed by the restoration of blood flow and reoxygenation. Insufficient blood supply leads to tissue hypoxia and cellular metabolic imbalance, and subsequent reperfusion and reoxygenation cause excessive inflammatory responses and exacerbate ischemic tissue damage, which is known as I/R injury [1]. I/R injury is intrinsically associated with oxidative damage, and multiple pathological processes contribute to this damage, including impaired endothelial barrier function, mitochondrial dysfunction, activation of the cell death program, calcium overload, sterile inflammation and autoimmune responses [2]. However, the precise molecular mechanism of I/R injury has not been fully elucidated and targeted therapies are still limited. Overall, therapeutic strategies for this condition need to be developed, and examining new therapeutic targets to manage I/R injury is a top priority.

Cell death is a stable pathological indicator of I/R injury. Emerging evidence has revealed a novel therapeutic concept to target regulated cell death (RCD) to counteract I/R injury, although the role of RCD in I/R injury has only recently become apparent [3]. Different forms of RCD have been identified in I/R injury, including autophagy, necroptosis, apoptosis, pyroptosis, parthanatos and ferroptosis [2]. Recent studies suggest that targeting RCD exerts beneficial effects against I/R injury; in particular, RCD in parenchymal and endothelial cells is recognized as a promising intervention target. I/R injury leads to RCD of parenchymal and endothelial cells, and apoptosis, necroptosis and autophagy are the most common types [4]. Generally, regulating I/R-induced RCD has been recognized as a new therapeutic strategy against I/R injury, but effective interventions are rarely summarized.

Recently, ferroptosis, a form of RCD characterized by iron-dependent lipid peroxidation, glutathione (GSH) depletion and glutathione peroxidase 4 (GPX4) inactivation [5], has received great attention in I/R events [6]. Ferroptosis occurs during the reperfusion but not the ischemic phase, as the levels of two key enzymes in ferroptosis, GPX4 and long-chain-fatty-acid-CoA ligase 4 (ACSL4) in tissues were significantly regulated only during reperfusion, accompanied by elevated iron concentration and malondialdehyde (MDA) levels [7]. During the reperfusion phase, mitochondrial respiration is enhanced, which consequently triggers reactive oxygen species (ROS) explosion and ferroptosis [8]. Notably, decreased mitochondrial membrane potential (MMP) can be observed, which indicates increased mitochondrial outer membrane permeability, a characteristic of mitochondrial-mediated apoptosis [9]. Although caspases are activated during this process, interventions targeting apoptosis do not completely prevent cell death. Thus, although activated in I/R, apoptosis is not essential for subsequent cell death, suggesting the existence of other mechanisms governing cell death [10], such as ferroptosis. And indeed, mitochondria play a much more active role in ferroptosis than in apoptosis (17). Moreover, some morphological characteristics of mitochondria during I/R, including reduced mitochondrial volume, reduced or even lost mitochondrial cristae, and condensed mitochondrial membrane densities, are not associated with other forms of cell death, further emphasizing the relevance of ferroptosis [8].

Ferroptosis has been implicated in the pathogenesis of I/R injury in multiple organs, using ferroptosis inhibitors, as well as targeting ferroptosis regulatory genes, which has been demonstrated to attenuate pathological damage in various I/R models [11] (Table 1 and Table 2). Inhibiting ferroptosis has the potential to become an effective therapeutic strategy for I/R injury. However, the regulatory mechanisms of ferroptotic death in I/R remain largely uncharacterized, and it is necessary to further examine the pathological mechanism and targeted therapeutic strategies of ferroptosis in I/R conditions. In the current review, we summarize the recent research progress on ferroptosis in I/R injury, including ferroptosis regulatory genes involved in I/R and the available evidence for the therapeutic application of ferroptosis regulators, hoping to reflect the state of the art and provide a direction for future trials.

## 2. Pathological Mechanism of Ferroptosis in I/R Injury

I/R injury is accompanied by various pathological processes, including increased intracellular iron concentrations and excessive ROS generation, which are accompanied by lipid peroxidation following reperfusion [87]. These events lead to cellular oxidative damage and are consistent with the characteristics of ferroptosis, a process in which iron-dependent ROS accumulation exceeds the ability of cells to maintain redox homeostasis, resulting in lipid peroxidation and ultimately cell death [5]. Notably, evidence suggested that iron and lipid peroxidation are necessary for the propagation of ferroptosis but not for cell rupture [88]. Take myocardial I/R as an example, given that ferroptosis occurs in myocardial I/R [21,56] and the increased intracellular iron concentrations as well as lipid peroxidation [64,89], this process may contribute significantly to the increased infarct size during reperfusion. Oxidative stress is the main cause of MIRI, which is due to enhanced ROS production. Excessive accumulation of ROS can cause membrane lipid peroxidation and disrupts the barrier function of the cell membrane. Moreover, the excessive oxidation of lipids and proteins together facilitates cardiomyocyte damage and eventually leads to cell death. During the reperfusion phase, the re-oxygenation of myocardial tissue with the recovery of blood flow will cause a sudden enhanced ROS production within the first few minutes, which is one of the pathogenic mechanisms of MIRI. In addition to lipid peroxidation, oxidative stress induced by the Fenton reaction is also of great significance for ferroptosis. Iron homeostasis imbalance increases intracellular free iron, which consequently improves hydroxyl radical (•OH) generation through the Fenton reaction. These reactive species cause cellular damage by attacking lipids, proteins and DNA while activating cell death pathways, including ferroptosis [90]. Additionally, excessive iron is transported into cells after I/R, accelerating the accumulation of ROS through the Fenton reaction and Haber–Weiss reaction, which ultimately makes cells more vulnerable to ferroptosis.

Ferroptosis is a biological process regulated by multiple genes and is regulated by a variety of cellular metabolic pathways, including iron metabolism, amino acid metabolism and lipid metabolism [91]. The morphological characteristics of ferroptosis are reduced mitochondrial volume, reduction of mitochondrial cristae and increased bilayer membrane density [5]. These changes are caused by high lipid peroxidation levels in the phospholipid pool of the cell membranes [88]. When polyunsaturated fatty acyls (PUFAs) exist in the phospholipid pool, it is highly susceptible to lipoxygenase (LOX)-driven peroxidation, for hydrogen extraction from PUFAs is prone to produce peroxyl radicals [92]. ACSL4 plays a key role in this process, as it expands the membrane lipids pool containing PUFAs, thereby increasing ferroptosis sensitivity [93]. Ferroptosis is iron-dependent, the enzyme activity of LOX requires iron to maintain [94], whereas iron can oxidize phospholipids in the Fenton reaction independent of LOX [95]. Generally, cells uptake iron through transferrin receptor 1 (TfR1) and then store iron in ferritin complexes. However, lysosomal and autophagic mechanisms can decompose these complexes to release iron and further sensitize them for ferroptosis in ferritinophagy [96]. These reactions consistently happen but typically do not cause cell death, as there are multiple intracellular antioxidative systems. The GPX4 system is the most prominent of these systems [97]. The enzyme x_c_-controls the cellular import of cystine to exchange glutamate at the plasma membrane, a necessary step for the production of the GPX4 substrate GSH. By oxidizing GSH, GPX4 can reduce peroxidized lipids, thus, inhibition of GPX4 or cystine import can induce ferroptosis [97]. In addition, coenzyme Q10 (CoQ10) can reverse lipid peroxidation independent of GPX4, ferroptosis suppressor protein 1 (FSP1) can act as an oxidoreductase of CoQ10, shuttling reductants to the lipid bilayer plasma membrane to protect against peroxidation damage [98]. Furthermore, mitochondrial membrane peroxidation can be reversed by dihydroorotate dehydrogenase (DHODH), which acts in parallel with GPX4 by reducing CoQ10 [99]. Another protective enzyme is GTP cyclohydrolase 1 (GCH1), which protects PUFA phospholipids from ferroptotic degradation independent of GPX4 and it can also reduce CoQ10 [100]. Collectively, ferroptosis occurs through plasma membrane pores caused by lipid peroxidation, but cells are equipped with various systems against lipid peroxidation. Targeting key molecules and processes in these systems is the basic route to inhibit ferroptosis and improve the prognosis of related diseases, including but not limited to I/R injury.

**Figure 1:**
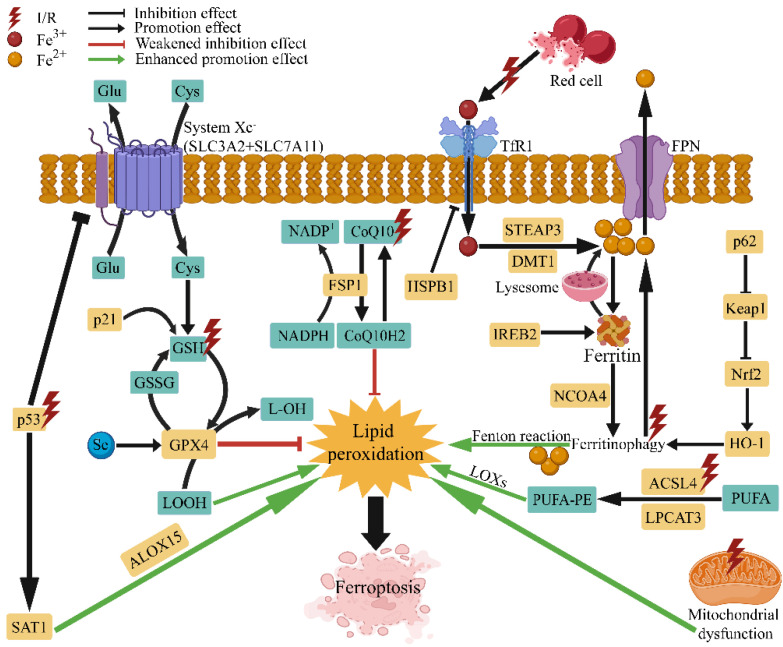
Overview of the regulatory mechanism of ferroptosis in ischemia-reperfusion. I/R, ischemia-reperfusion; Glu, glutamate; Cys, cystine; SLC3A2, solute carrier family 3 member 2; SLC7A11, solute carrier family 7 member 11; p21, cyclin dependent kinase inhibitor 1A; p53, protein 53; GSH, glutathione; GSSG, glutathione oxidized; Se, selenium; GPX4, glutathione peroxidase 4; SAT1, spermidine/spermine N1-acetyltransferase 1; ALOX-15, arachidonate lipoxygenase 15; NADPH, nicotinamide adenine dinucleotide phosphate; CoQ10, coenzyme Q10; FSP1, ferroptosis suppressor protein 1; HSPB1, heat shock factor-binding protein 1; TfR1, transferrin receptor 1; FPN, ferroportin; STEAP3, six transmembrane epithelial antigen of the prostate 3; DMT1, divalent metal-ion transporter-1; IREB2, iron response element-binding protein 2; NCOA4, nuclear receptor coactivator 4; LOXs, lipoxygenases; PUFA, polyunsaturated fatty acids; PUFA-PE, polyunsaturated phosphatidylethanolamines; ACSL4, acyl-CoA synthetase long-chain family member 4; LPCAT3, lysophosphatidylcholine acyltransferase 3; HO-1, heme oxygenase 1; Nrf2, nuclear factor erythroid 2-related factor 2; Keap1, kelch-like ECH-associated protein 1; p62, sequestosome 1.

There is long-standing evidence that targeting iron is a potential therapeutic strategy for I/R injury. Previous clinical studies have shown that iron levels are significantly increased in regions of the brain with severe ischemia-hypoxia [101]. Additionally, I/R-induced increases in iron levels have been associated with exacerbated tissue damage [12], and modulating iron homeostasis has been shown to reduce I/R injury [102]. Moreover, free iron is necessary for the initiation of ferroptosis. There is a labile iron pool (LIP) in the cytoplasm, lysosomes and mitochondria [103], and free iron released from the LIP during I/R accelerates lipid peroxidation and ferroptosis through the Fenton reaction [104]. On the other hand, reperfusion of ischemic tissues leads to excessive ROS generation that exacerbates I/R injury [105], and various antioxidants have been shown to protect against I/R injury [106]. Notably, nonenzymatic production of ROS is promoted when metal ions are present, such as iron, which is involved in ROS production in mitochondria. Evidence suggests that ferroptosis occurs during the reperfusion period rather than the ischemic period [7]. Mitochondrial dysfunction during ischemia leads to excessive ROS levels, which cannot be effectively scavenged and exacerbate ferroptosis together with lipid peroxidation following reperfusion [87]. Therefore, I/R injury, which is mainly characterized by oxidative damage and accompanied by the dysregulation of iron homeostasis, is inseparable from ferroptosis (Figure 1). Since the beginning of ferroptosis-related research, many ferroptosis inducers and inhibitors have been identified, such as erastin, RSL3, sulfasalazine, sorafenib, ferrostatin-1 (Fer-1) and liproxstatin-1 (Lip-1). Additionally, ferroptosis can be inhibited by iron chelators, peroxidation inhibitors and antioxidants [107], and most of these agents have been used to further elucidate the mechanism by which ferroptosis is involved in I/R injury. Ferroptosis has multiple regulatory genes, such as GPX4, ACSL4, solute carrier family 7 member 11 (SLC7A11), nuclear factor erythroid 2-related factor 2 (Nrf2), nuclear receptor coactivator 4 (NCOA4), and heme oxygenase 1 (HO-1). These genes are involved in multiple mechanisms related to ferroptosis, such as iron metabolism, oxidative stress and lipid peroxidation, and targeted therapeutic strategies associated with these genes have also been investigated in various I/R models [108]. Collectively, the important role of ferroptosis in mediating I/R injury has added a new impetus to the treatment of I/R injury and initiated a wave of developing novel therapeutic strategies for I/R injury.

## 3. Therapeutic Strategies Targeting Ferroptosis in I/R Injury

### 3.1. Ferroptosis in Cerebral I/R Injury

Cerebral infarction, which is also known as ischemic stroke, is an episode of neurological dysfunction induced by the focal cerebral, spinal cord, or retinal infarction [109]. Previous studies suggested that ischemic stroke was complicated by iron accumulation in the affected regions, which exacerbated neuronal damage during reperfusion [110]. Moreover, iron overload is the major source of oxidative stress in cerebral I/R [111], and an adverse prognosis in patients with cerebral ischemia has been associated with elevated brain iron levels [112]. Similarly, animals treated with a high-iron diet were more susceptible to middle cerebral artery occlusion (MCAO) [113], and iron chelation therapy attenuated I/R injury [114].

#### 3.1.1. Therapeutic Targets of Ferroptosis in Cerebral I/R Injury

Iron is an important driver of lipid peroxidation and ferroptosis [96]. Tau protein facilitates neuronal iron efflux, the loss of which may result in neurotoxic iron accumulation [115]. Tuo et al. [12] found that MCAO suppressed tau and increased iron levels in 3-month-old rats and mice, whereas tau knockout did not increase brain iron levels in these mice and protected the hemispheres from I/R injury. However, the protective effect was counteracted and there was accelerated iron accumulation in the brains of aged tau-knockout mice until iron-targeted intervention restored it. Additionally, the ferroptosis inhibitors Fer-1 and Lip-1 attenuated neurological deficits following cerebral I/R. These findings suggest that the tau-iron interaction is a pleiotropic regulator of ferroptosis and cerebral I/R injury. As another key component of iron homeostasis, ferritin has been shown to protect against oxidative injury [116], and its degradation has also been demonstrated to trigger ferroptosis [117]. A recent study reported that ferritin overexpression exerted a neuroprotective effect against MCAO-induced oxidative hippocampal neuronal death [13]. Notably, the increases in p53 and SLC7A11 expression were reversed by ferritin overexpression, suggesting that I/R-induced ferroptosis was suppressed. In addition to cytosolic ferritin, mitochondrial ferritin (FtMt) has also been associated with iron-dependent oxidative damage and ferroptosis [118]. In cerebral I/R injury, ferroptosis activation was accompanied by FtMt upregulation [15]. FtMt-knockout mice exhibited worsened neurological deficits with typical ferroptosis features after cerebral I/R. Conversely, FtMt overexpression reversed ferroptosis activation and consequently ameliorated cerebral I/R injury. Ferritinophagy is a process in which ferritin is transported to lysosomes for degradation [119]. NCOA4 has been demonstrated to promote ferritinophagy [120] and further induces ferroptosis [121]. Recently, the involvement of NCOA4-mediated ferritinophagy was identified in cerebral I/R, and NCOA4 silencing significantly inhibited ferroptosis and prevented neuronal damage [14]. Moreover, NCOA4 was reported to be upregulated by ubiquitin-specific peptidase 14 (USP14) in damaged neurons, and USP14 inhibition effectively decreased NCOA4 levels to suppress ferritinophagy-mediated ferroptosis. These findings provide evidence that targeting ferroptosis by regulating iron metabolism-related proteins/pathways is a potential strategy for ischemic stroke treatment.

In addition to targeting iron metabolism-related genes, strategies to modulate oxidative stress-related mechanisms to suppress ferroptosis in cerebral I/R have also been investigated. UbiA prenyltransferase domain-containing 1 (UBIAD1) is an antioxidant enzyme that catalyzes the biosynthesis of coenzyme Q10 (CoQ10) in the Golgi apparatus membrane [122]. In nonmitochondrial systems, CoQ10 is involved in regulating lipid peroxidation, paralleling the GPX4 pathway in ferroptosis [98]. Huang et al. [16] demonstrated that UBIAD1 was upregulated during cerebral I/R, which suppressed lipid peroxidation and ferroptosis and exerted neuroprotective effects. UBIAD1 alleviated cerebral I/R-mediated ferroptotic neuronal death by enhancing antioxidant capacities by ameliorating mitochondria and Golgi apparatus dysfunction, suggesting that restoring impaired mitochondria and the Golgi apparatus is beneficial in ameliorating cerebral I/R injury. The cyclooxygenase 2 (COX-2)/prostaglandin E2 (PGE2) pathway is known to be involved in cerebral I/R, and evidence suggests an association between ferroptosis and the COX-2/PGE2 pathway [123]. Xu et al. [17] found that inhibiting cerebral I/R-induced ferroptosis inactivated PGE2 synthases, degraded enzymes and PGE2 receptors and reduced cerebral infarct size. Conversely, PGE2 could suppress ferroptosis by inhibiting iron accumulation and lipid peroxidation. Polyamine imbalance has been reported in cerebral I/R injury, and inhibiting spermine metabolism to putrescine may prevent neuronal I/R injury [124]. Spermidine/spermine N1-acetyltransferase 1 (SAT1) is a rate-limiting enzyme in cellular polyamine metabolism that facilitates the conversion of spermidine and spermine to putrescine [125]. Zhao et al. [18] investigated the role of the SAT1/arachidonate 15-lipoxygenase (ALOX15) axis in neuronal ferroptosis after cerebral I/R. The researchers demonstrated that *SAT1* knockdown reduced ROS levels and cortical iron levels in cerebral I/R and further attenuated neurological injury. Collectively, activation of the SAT1/ALOX15 axis exacerbated cerebral I/R injury by triggering neuronal ferroptosis, and inhibition of SAT1 alleviated cerebral I/R injury. As important contributors to ferroptosis, ACSL4 genes/proteins, as well as their phosphatidylethanolamine lipid products, were significantly altered in response to MCAO [19]. Moreover, thrombin was shown to induce ferroptotic signaling by promoting arachidonic acid mobilization and the subsequent esterification of ACSL4, suggesting that targeting the thrombin/ACSL4 axis may alleviate ischemic stroke via ferroptosis inhibition.

microRNA (miRNA) function in various pathophysiological processes, including I/R-induced cell death [126], and the role of miRNAs in regulating ferroptosis has been demonstrated in cancers [104]. Similarly, lncRNAs play critical regulatory roles in multiple biological processes and diseases, including I/R injury [127]. Ischemic stroke has been shown to change miRNAs and long noncoding RNA (lncRNA) expression profiles [128,129]. For instance, elevated miR-214 exerted antioxidant and antiapoptotic effects during I/R injury [130]. Inspired by the altered plasma levels of lncRNA PVT1 (plasmacytoma variant 1) and miR-214 in ischemic stroke patients, Lu et al. [20] investigated the therapeutic effects of targeting PVT1 and miR-214 in cerebral I/R models. PVT1 silencing and miR-214 overexpression reduced infarct size and, importantly, suppressed ferroptosis in mice subjected to cerebral I/R. Furthermore, PVT1 overexpression or miR-214 silencing abolished the effects of Fer-1 on ferroptosis indicators. These results suggest the possibility of targeting PVT1 and miR-214 for ischemic stroke treatment.

#### 3.1.2. Pharmacological Therapies Targeting Ferroptosis in Cerebral I/R Injury

Accumulating evidence suggests that pharmacological interventions inhibiting ferroptosis prevent I/R-induced neuronal damage [6]. Since being identified as a master regulator of ferroptosis [131], selenium-dependent GPX4 has been associated with brain diseases, including ischemic stroke [107]. Alim et al. [48] reported that selenium (Se) drove a transcriptional adaptive response to prevent ferroptosis and alleviate hemorrhagic or ischemic stroke. Pharmacological selenium administration augmented GPX4 via coordinated activation of transcription factor AP-2 gamma (TFAP2c) and special protein 1 (Sp1) to inhibit ferroptosis and exert neuroprotective effects, providing a new strategy for stroke management. Recently, the anti-ferroptotic effects of several selenium compounds were characterized in murine models of MCAO [49,50], further supporting the strategy of using pharmacological selenium to prevent cerebral I/R injury. Some agents with antioxidant or neuroprotective effects have also been reported to inhibit ferroptosis and reduce cerebral I/R injury. Carvacrol (CAR) is a monoterpene phenol with antiproliferative, antiapoptotic, and neuroprotective properties [132]. Guan et al. [51] demonstrated that CAR protected against I/R-induced hippocampal neuronal impairment by mitigating ferroptosis by increasing the expression of GPX4, providing new insights into the mechanism of CAR-mediated neuroprotective effects on cerebral I/R injury. Rehmannioside A [52], bioflavonoids including galangin [53], carthamin yellow [54] and kaempferol [55], have been shown to ameliorate I/R-induced neuronal ferroptosis by upregulating the SLC7A11/GPX4-related axis or inhibiting ACSL4 expression. These findings provide valuable insight into the pathogenesis and available agents for treating ischemic stroke.

### 3.2. Ferroptosis in Myocardial I/R Injury

Acute myocardial infarction (AMI) accounts for a large proportion of global mortality [133]. Although effective myocardial reperfusion strategies are essential for ischemic tissue survival, reperfusion itself may trigger cardiomyocyte dysfunction, which is known as MIRI [134]. Emerging evidence suggests that dysregulation of iron homeostasis is involved in the pathogenesis of AMI. Following I/R, excess iron is transported into cells and predisposes cardiomyocytes to undergo ferroptosis via the Fenton reaction and ROS accumulation [135]. Precisely targeting ferroptosis has been suggested to be a promising therapeutic option for reversing MIRI, and multiple ferroptosis inhibitors and ferroptosis-regulated genes involved in the MIRI setting have been reported [136].

#### 3.2.1. Therapeutic Targets of Ferroptosis in Myocardial I/R Injury

Ferroptosis is closely associated with cellular metabolism and redox machinery. The serum factors transferrin and glutamine have been previously shown to be ferroptosis inducers. Gao et al. [21] demonstrated that transferrin and intracellular glutaminolysis are essential for the execution of ferroptosis. The glutaminolysis inhibitor Compound 968 and the iron chelator deferoxamine (DFO) mitigated I/R injury and improved cardiac function in mice, suggesting that targeting glutaminolysis to suppress ferroptosis is a potential therapy for MIRI. As a member of the deubiquitinase family, ubiquitin-specific protease 22 (USP22) has been reported to direct the stabilization of sirtuin-1 (SIRT1) to inhibit p53 transcriptional activity and proapoptotic functions [137]. Recently, the USP22/SIRT1 axis was shown to play a pivotal role in ferroptosis-mediated I/R-induced cardiomyocyte damage [22]. Increases in USP22, SIRT1, or SLC7A11 attenuated ferroptosis and ameliorated cardiac function, which was accompanied by reduced ROS production, lipid peroxidation and iron accumulation. Intriguingly, in contrast to USP22, ubiquitin-specific protease 7 (USP7) was reported to promote ferroptosis in MIRI by activating the p53/TfR1 pathway [23].

As the only known mammalian iron-exporting protein, ferroportin 1 (FPN1) plays a key role in systemic iron homeostasis, and hepcidin-mediated internalization and degradation of FPN1 are critical for maintaining cardiac iron homeostasis [138]. As a key regulator of FPN1 transcription [139], Nrf2 has been shown to alleviate myocardial injury by inhibiting I/R-induced oxidative damage and cell death [140]. Modulation of the Nrf2/FPN1 signaling pathway was shown to be a promising strategy to restrict ferroptosis and alleviate diabetic myocardial reperfusion injury in a recent study [24]. Similar to cerebral I/R injury [14], ferritinophagy contributes to I/R-induced ferroptosis. DNA (cytosine-5)-methyltransferase 1 (DNMT-1) was shown to affect ferroptosis in diabetic myocardial (DM) I/R by regulating NCOA4-mediated ferritinophagy [25]. The increase in ferroptosis was accompanied by elevated DNMT-1 and NCOA4 levels in myocardial tissues in the DM I/R group, while a DNMT-1 inhibitor reversed NCOA4-mediated ferritinophagy and myocardial injury. Moreover, DNMT-1 inhibition enhanced the protective effect of NCOA4-siRNA in a cell H/R model. Therefore, inhibiting DNMT-1 may be a therapeutic strategy for reducing ferroptosis during myocardial I/R by regulating NCOA4-mediated ferritinophagy.

Fragmented oxidized phosphatidylcholines (OxPCs) have been reported to induce cell death in neonatal cardiomyocytes, and several types of OxPCs are increased in models of MIRI [141]. Stamenkovic et al. [26] recently demonstrated that OxPCs were generated during I/R disrupted mitochondrial bioenergetics and calcium transients and could induce cardiomyocyte death. Notably, GPX4 activity was suppressed in isolated cardiomyocytes treated with OxPCs, which was associated with increased ferroptosis. Furthermore, the neutralization of OxPCs prevented ferroptosis during I/R. Thus, interventions targeting OxPCs may mitigate MIRI. Embryonic lethal-abnormal vision-like protein 1 (ELAVL1) is an RNA-binding protein that regulates gene expression by stabilizing mRNAs [142]. Previous studies have suggested its role in pyroptosis in diabetic hearts [143] and in ferroptosis during liver fibrosis [144]. Chen et al. [27] demonstrated that Forkhead Box C1 (FOXC1) transcriptionally activated ELAVL1, increased I/R-induced autophagic ferroptosis, and exacerbated the myocardial injury. ELAVL1 knockdown significantly suppressed ferroptosis and ameliorated pathological damage in mice undergoing myocardial I/R surgery, indicating that ELAVL1 may serve as a target for suppressing I/R-induced ferroptosis.

In addition to those in ischemic stroke, the roles of several miRNAs and lncRNAs in regulating ferroptosis have also been reported in myocardial I/R. MiR-135b-3p is an oncogene that accelerates tumor development [145]. Recently, this factor was shown to exert an inhibitory effect on GPX4 expression, thereby exacerbating ferroptosis during MIRI [28]. Accumulating evidence suggests that lncRNAs can act as competing endogenous RNAs (ceRNAs) and sponge miRNAs to upregulate downstream gene expression [146]. LncAABR07025387.1 was shown to function as a ceRNA to sponge miR-205 (downregulating miR-205 expression) and consequently enhanced ACSL4 expression to exacerbate ferroptosis during myocardial I/R [29]. In contrast, several lncRNAs are cardioprotective against I/R injury. The bone marrow mesenchymal stem cell (BMSC)-derived lncRNA Mir9-3hg, which has been previously reported to suppress bladder cancer progression [147], can suppress I/R-induced cardiomyocyte ferroptosis by regulating the pumilio RNA binding family member 2 (Pum2)/peroxiredoxin 6 (PRDX6) axis [30]. These findings highlight the therapeutic potential of using functional noncoding RNAs to treat MIRI by modulating ferroptosis.

#### 3.2.2. Pharmacological Therapies Targeting Ferroptosis in Myocardial I/R Injury

Pharmacological therapeutic strategies targeting ferroptosis inhibition in MIRI have also been developed. As a well-established ferroptosis inhibitor, the effect of Lip-1 on reducing cell death in the ischemic myocardium has been investigated in a murine model of MIRI [56]. The cardioprotective effect of Lip-1 is mainly achieved by abrogating the I/R stress-induced reduction in GPX4 and inhibiting mitochondrial ROS generation. Similarly, other well-recognized ferroptosis inhibitors, such as Fer-1 [59], DFO [63] and dexrazoxane (DXZ) [64], have been shown to provide effective protection against MIRI by attenuating ferroptosis. However, it should be noted that even some common ferroptosis inhibitors, such as DXZ, an intracellular metal chelator, have previously been reported to have no protective effect on MIRI in pigs [148]. Thus, the different pharmacodynamics, bioavailability and subcellular localization of ferroptosis inhibitors should be considered in future studies on I/R injuries.

Ferroptosis is characterized by iron-dependent accumulation of lipid peroxides, which leads to oxidative damage to lipid bilayers. Thus, preventing oxidative stress is a common strategy to alleviate ferroptotic injury, and a variety of ferroptosis inhibitors that prevent oxidative stress have been developed and validated in I/R models. Gossypol acetic acid (GAA), a natural constituent extracted from the seeds of cotton plants, has been shown to inhibit oxidative effects and lipid peroxidation in rat hepatic tissues [149]. Additionally, the iron-chelating effect of GAA has been confirmed [150]. It was recently reported that GAA was beneficial in myocardial I/R by protecting cardiomyocytes against ferroptosis and reducing infarct size [65]. Histochrome (HC) is a water-soluble form of echinochrome A with strong iron-chelating and antioxidant properties [151]. As a clinically available drug, HC has been previously shown to alleviate MIRI by reducing arrhythmia potential and infarct size [152]. Hwang et al. [66] recently found that the administration of HC protected the myocardium from I/R injury by suppressing ferroptosis, as indicated by a reduction in ROS levels, the maintenance of GSH levels, and the upregulation of GPX4 activity. As a member of the anthocyanin family, cyanidin-3-glucoside (C3G) has antioxidant and anti-inflammatory properties [153]. The cardioprotective effects of C3G have also been reported in doxorubicin-induced cardiotoxicity and diastolic heart dysfunction [154,155]. In a rat model of MIRI, C3G administration attenuated oxidative stress and inhibited the expression of ferroptosis-related proteins, thereby suppressing ferroptosis and mitigating pathological cardiac damage [67], indicating that C3G is a potential agent to protect the myocardium from I/R injury. Flavonoids can inhibit lipid peroxidation and chelate redox-active metals and have been shown to attenuate myocardial infarction [156]. Moreover, it was reported that flavonoids could inhibit ferroptosis in human pancreatic ductal adenocarcinoma [157]. Xanthohumol (XN), a prenylated flavonoid extracted from *Humulus lupulus*, exerts various pharmacological effects, including inhibiting lipid peroxidation [158]. Recent evidence suggests that XN can protect cardiomyocytes against ferroptosis by inhibiting lipid peroxidation and ROS generation, chelating iron and modulating Nrf2 and GPX4 [68]. A considerable number of compounds with antioxidant activity can ameliorate I/R-induced ferroptosis, and exploiting these properties could provide new strategies for the management of those with myocardial infarction.

The Nrf2 signaling pathway has received extensive attention in ferroptosis-related studies. Several compounds or clinically used drugs have been reported to reduce I/R-induced ferroptosis and myocardial damage by regulating Nrf2 signaling. Etomidate (Eto), a short-acting anesthetic that has been previously shown to ameliorate I/R injury [159], was reported to attenuate MIRI by inhibiting ferroptosis through the Nrf2/HO-1 pathway [69]. Naringenin (NAR), a flavonoid-rich in various biological activities, has significant effects on improving I/R injury [160]. Xu et al. [70] recently concluded that NAR could alleviate myocardial I/R-induced pathological damage by inhibiting ferroptosis via the Nrf2/SLC7A11/GPX4 axis. Britanin (Bri) is a sesquiterpene lactone extracted from *Inula linariifolia* with excellent antioxidative and anti-inflammatory activities [161]. Bri has been reported to protect against cerebral I/R injury by regulating the Nrf2 signaling pathway [162]. In a recent study on MIRI, Bri was shown to exert a protective effect against ferroptosis-mediated myocardial I/R damage by upregulating GPX4 expression through the activation of adenosine monophosphate-activated protein kinase (AMPK)/glycogen synthase kinase 3β (GSK3β)/Nrf2 signaling [71]. The effects of these agents verified the importance of targeting the Nrf2 pathway to alleviate ferroptosis-mediated I/R injury.

Apart from these drugs/compounds, a variety of agents with different biological activities have been shown to inhibit ferroptosis in MIRI. Propofol, a frequently used anesthetic agent, has been shown to protect against myocardial injury by suppressing I/R-induced ferroptosis through the AKT/p53 signaling pathway [72]. Ferulic acid (FA), the main active component of *Angelica sinensis*, was reported to alleviate MIRI by enhancing AMPKα2 expression-mediated ferroptosis inhibition [73]. Polydopamine nanoparticles (PDA NPs), a new form of ferroptosis inhibitor with favorable biocompatibility and biodegradability, were shown to effectively reduce iron deposition and lipid peroxidation in a murine model of MIRI [74]. UAMC-3203, a novel ferroptosis inhibitor modified from Fer-1 analogs, inhibited ferroptosis and significantly attenuated I/R myocardial damage [75]. Resveratrol (Res), a polyphenol with multiple biological activities, was shown to alleviate MIRI by inhibiting ferroptosis through the regulation of ubiquitin-specific peptidase 19 (USP19)-Beclin 1-mediated autophagy [76]. Dexmedetomidine (DEX), a selective α2-adrenergic receptor, was shown to alleviate myocardial damage by inhibiting ferroptosis through the promotion of the SLC7A11/GPX4 axis [77]. Collectively, alternative anti-ferroptosis agents continue to emerge, but the specific mechanisms and drug safety need to be further evaluated in disease models, including but not limited to I/R injury.

### 3.3. Ferroptosis in Lung I/R Injury

Although research remains scarce, recent evidence shows that ferroptosis is involved in lung I/R injury (LIRI) and can be modulated to mitigate lung damage. Xu et al. [57] first demonstrated the involvement of ferroptosis in LIRI, and GPX4 and ACSL4 were significantly regulated in lung I/R conditions. Consistent with the findings in other I/R models, increased tissue iron levels, lipid peroxidation and ferroptosis-like mitochondrial morphological changes were observed in the hilar clamp murine model of LIRI. Moreover, the administration of rosiglitazone (ROSI) inhibited ACSL4 expression, decreasing lung I/R-induced ferroptotic damage. Consistent with the findings in intestinal ischemia-reperfusion-induced acute lung injury (IIR-ALI) [47], pretreatment with Lip-1 suppressed inflammation and attenuated lung injury following I/R. Irisin is a hormone-like molecule that is mainly secreted by skeletal muscles during exercise, and its anti-inflammatory, antioxidative and antiferroptotic activities have attracted much attention [163]. Irisin has been reported to prevent LIRI by restoring mitochondrial function [60]. Recently, irisin treatment prevented I/R lung damage by suppressing ferroptosis through the Nrf2/HO-1 axis [60], which was similar to the effect of Fer-1. These findings indicate the involvement of ferroptosis in the progression of LIRI. Because of the critical role of oxidative stress in LIRI, inhibiting ferroptosis to attenuate oxidative damage might be a new strategy for preventing I/R lung injury.

### 3.4. Ferroptosis in Hepatic I/R Injury

#### 3.4.1. Therapeutic Targets of Ferroptosis in Hepatic I/R Injury

Hepatic I/R injury, which commonly occurs during liver surgical procedures, can induce severe liver damage and different forms of cell death, including ferroptosis. A high serum ferritin level in donors is a risk factor for I/R injury in liver transplantation [61], and evidence indicates that ferroptosis may be a promising therapeutic target in hepatic I/R injury [164]. Not surprisingly, most ferroptosis regulatory targets that have been studied in hepatic I/R are related to iron metabolism. Macrophages play a central role in regulating iron homeostasis and participate in hepatic I/R injury. Macrophage extracellular trap (MET) formation and ferroptosis were more severe both in patients who underwent hepatectomy and mice subjected to hepatic I/R [31]. Intriguingly, I/R-induced ferroptosis was reversed by inhibiting MET formation. Additionally, Fer-1 or DFO administration attenuated I/R-induced liver injury, which highlighted the therapeutic potential of inhibiting MET formation and ferroptosis in reversing hepatic I/R injury. The HECT domain-containing ubiquitin E3 ligase (HUWE1) was initially shown to regulate apoptosis [165]. However, little is known about the role of HUWE1 in pathological conditions with high levels of cell death, such as hepatic I/R. Wu et al. [32] reported an association between high expression of HUWE1 and reduced hepatic injury in liver transplantation patients and further identified HUWE1 as a novel negative ferroptosis modulator that targets TfR1 for ubiquitination and proteasomal degradation to regulate iron metabolism. IREB2 plays a central role in cellular iron homeostasis [166] and has been suggested to be a marker gene for ferroptosis [5]. Li et al. [33] recently reported increased IREB2 expression in a steatotic liver I/R model, and *Ireb2* knockdown significantly reduced iron levels and suppressed ferroptosis. Furthermore, miR-29a-3p could downregulate IREB2 expression and inhibit ferroptosis, and the downregulation of miR-29a-3p abolished this protective effect. Fatty livers are extremely sensitive to I/R injury and poorly tolerate this condition, and the authors concluded that targeting Ireb2 via exosomal transfer of miR-29a-3p was a potential preventive strategy.

#### 3.4.2. Pharmacological Therapies Targeting Ferroptosis in Hepatic I/R Injury

To date, few ferroptosis inhibitors have been investigated in hepatic I/R models, and the agents that have been initially validated in other I/R models have not yet been examined in hepatic I/R models. Friedmann Angeli et al. [58] discovered that Lip-1 suppressed ferroptosis and reversed liver injury in a preclinical model of hepatic I/R. Similarly, pretreatment with Fer-1 or α-tocopherol (the active form of vitamin E) significantly prevented hepatic I/R-induced ferroptosis-mediated pathological damage [61]. Hepatic I/R injury was also attenuated by the iron chelator DFO but was exacerbated in mice that were fed a high-iron diet [61]. These results suggest the important role of ferroptosis in the pathogenesis of hepatic I/R injury.

### 3.5. Ferroptosis in Renal I/R Injury

#### 3.5.1. Therapeutic Targets of Ferroptosis in Renal I/R Injury

Renal I/R injury remains a challenge in perioperative medicine and is closely associated with oxidative cell death. As demonstrated in murine models of I/R-induced AKI, ferroptosis causes synchronized necrosis in renal tubular cells. In addition to the known key enzymes associated with ferroptosis, recent studies have suggested some potential therapeutic targets for I/R-induced ferroptosis, which vary in the mechanisms by which they regulate ferroptosis. Augmenter of liver regeneration (ALR), a widely distributed multifunctional protein, has antioxidant properties [167]. Huang et al. [34] showed that silencing ALR exacerbated ferroptosis and increased ROS and mitochondrial damage in an in vitro model of I/R-induced AKI. Notably, the researchers demonstrated that ALR mediated ferroptosis in renal I/R injury and was linked to the glutathione-glutathione peroxidase (GSH-GPX) system. Pannexin 1 (Panx1), which is a protein involved in the ATP-release pathway, has a proapoptotic effect on AKI [168]. Su et al. reported that Panx1 contributed to ferroptosis-mediated renal I/R injury. Panx1 deletion decreased plasma creatinine and MDA levels and tubular cell death in the kidneys of mice subjected to renal I/R. Moreover, silencing Panx1 expression significantly attenuated iron accumulation and lipid peroxidation induced by the ferroptosis inducer erastin in cultured human kidney 2 (HK2) cells [35]. As we mentioned previously, ELAVL1 is an important regulator of ferroptosis and has been shown to increase myocardial I/R-induced autophagic ferroptosis [27]. Sui et al. [36] reported an interaction between ELAVL1 and cold-inducible RNA-binding protein (CIRBP). CIRBP expression was elevated in response to H/R or erastin in HK2 cells, and anti-CIRBP treatment suppressed ferroptosis and improved renal I/R injury. Therefore, CIRBP may promote ferroptosis in renal I/R injury. Legumain, an asparaginyl endopeptidase expressed in proximal tubular cells, is required for the maintenance of kidney homeostasis [169]. Legumain deficiency was recently shown to attenuate ferroptosis and tubular injury in renal I/R [37]. Mechanistically, legumain participates in the pathogenesis of I/R-induced AKI by regulating the degradation of GPX4, a major ferroptosis-protective factor. Indoleamine 2,3-dioxygenase 1 (IDO) is a rate-limiting enzyme that degrades tryptophan, and inhibiting IDO has been shown to preserve renal function in I/R injury [170]. Moreover, IDO expression was upregulated by anoxia and reoxygenation in a H/R model of primary renal proximal tubular epithelial cells (RPTECs) [38], which further induced aryl-hydrocarbon receptor (AhR)-mediated ferroptosis and exacerbated H/R damage. Lysine-specific demethylase 1 (LSD1) has been shown to regulate the pathogenesis of kidney disease. In renal I/R injury, LSD1 promotes oxidative stress and ferroptosis [39]. However, LSD1 inhibition blocked I/R-induced ferroptosis and oxidative stress by downregulating the TLR4/NOX4 pathway, suggesting that LSD1 is a potential therapeutic target for renal I/R injury.

Several miRNAs have also been identified as important regulators of I/R-induced renal injury. For instance, miR-378a-3p was significantly increased in the urine of rats subjected to renal I/R surgery [171]. MiR-182-5p was elevated in posttransplantation AKI patients [172], and miR-182-5p inhibition ameliorated I/R-induced renal injury [173]. MiRNAs can play functional roles by cooperating with other noncoding RNAs [174]. Ding et al. [40] found that miR-182-5p and miR-378-3p expression levels were upregulated in H/R-induced injury. Importantly, these two upregulated miRNAs jointly promoted the activation of ferroptosis by downregulating the expression of SLC7A11 and GPX4 [40]. Ferroptosis can be regulated by HO-1, which controls the iron level during ferroptotic death [175]. Inhibiting miR-3587 (a putative regulator of HO-1) was reported to upregulate HO-1 expression and protect renal cells against I/R-induced ferroptosis [41]. These studies provide new insights into targeting I/R-induced ferroptosis to alleviate renal I/R injury.

#### 3.5.2. Pharmacological Therapies Targeting Ferroptosis in Renal I/R Injury

Out of the concern for the potential metabolic and plasma instability of Fer-1, Linkermann et al. [80] generated third-generation ferrostatin 16–86 to suppress ferroptosis in renal I/R, which exerted a protective effect even under conditions with severe I/R injury and provided enhanced protection as a combination therapy with mitochondrial permeability transition inhibition. Increasing evidence suggests that pharmacological inhibition of ferroptosis is a promising therapeutic strategy to mitigate renal I/R injury. Inhibitors of ferroptosis, such as XJB-5-131 [81], quercetin [82], Nec-1f [83], Fer-1 and DFO [62], have been reported to suppress ferroptosis, thereby attenuating renal injury under I/R conditions. Therefore, postischemic renal necrosis may be targeted by ferrostatin therapy.

Instead of using ferroptosis inhibitors, other pharmacological treatments have been shown to prevent I/R-induced ferroptosis and protect against renal I/R injury. Consistent with the findings in LIRI [60], irisin treatment has been proven to alleviate tissue damage in a murine model of renal I/R by upregulating GPX4 expression [78]. Pachymic acid (PA), a lanostane-type triterpenoid isolated from *Poria cocos*, was shown to ameliorate renal damage in a murine model of renal I/R, which may be related to ferroptosis inhibition in the kidney via the upregulation of the Nrf2/HO-1 axis [79]. Entacapone, a specific inhibitor of catechol-O-methyltransferase (COMT), has long been used as an adjuvant therapy for Parkinson’s disease. Yang et al. [84] recently found that entacapone reversed ferroptosis and alleviated AKI by inhibiting lipid peroxidation and iron accumulation, and the underlying mechanism involves the upregulation SLC7A11 to enhance antioxidant capacity. These findings expand the uses of several existing agents and provide new strategies for the treatment of I/R-AKI.

### 3.6. Ferroptosis in Intestinal I/R Injury

Some researchers have focused on I/R-induced intestinal injury as well as IIR-induced remote tissue damage. Li et al. [42] demonstrated that suppressing ferroptosis with Lip-1 ameliorated I/R-induced intestinal injury. Additionally, inhibiting ACSL4 with ROSI before reperfusion prevented IIR injury, which indicates the role of ferroptosis in IIR. Consistent with findings in cerebral I/R injury [97], Sp1 was identified as a critical factor that promoted ACSL4 expression, which could be targeted to alleviate IIR-induced ferroptosis [42]. Apigenin-7-O-β-D-(-6″-p-coumaroyl)-glucopyranoside (APG), a flavonoid glycoside extracted from Clematis tangutica, has a strong antioxidant effect on I/R injuries [176]. Feng et al. [85] demonstrated that APG protected against endothelial ferroptosis and IIR injury by inhibiting MAO-B activation, attenuating ROS generation and decreasing iron accumulation. As important components of the gut, the roles of the gut microbiota and metabolites in I/R-induced ferroptosis and intestinal injury remain unclear. Deng et al. [43] reported that the gut microbiota metabolite capsiate (CAT) inhibited ferroptosis during IIR by increasing GPX4 expression through the activation of transient receptor potential cation channel subfamily V member 1 (TRPV1), which provides a potential strategy for the prevention of intestinal I/R damage. Not only in LIRI but the role of ferroptosis has also been studied in the murine model of IIR-ALI. Typical characteristics of ferroptosis, including increased lipid peroxide, decreased reduced GSH and mitochondrial morphological changes were observed in lung tissues of IIR groups. Furthermore, regulation of ferroptosis through the Nrf2-SLC7A11/HO-1 axis [44], Nrf2/STAT3/SLC7A11 axis [45], Nrf2/TERT/SLC7A11 axis [46], as well as an inhibitor of apoptosis-stimulating protein of p53 (iASPP) [47], isoliquiritin apioside (IA) [86], have been reported to alleviate IIR-induced lung injury.

## 4. Conclusions and Perspectives

**Figure 2:**
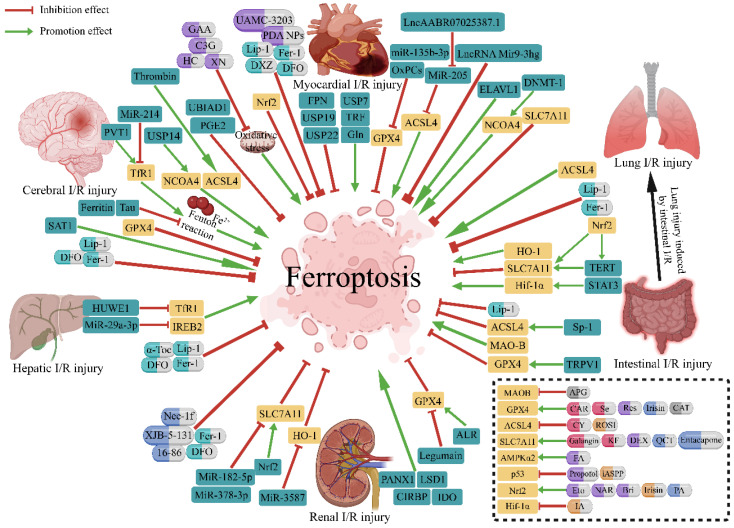
Therapeutic strategies targeting ferroptosis in ischemia-reperfusion injury. Regulatory genes and pharmacological therapies of ferroptosis investigated in I/R injury are summarized in the figure. Green capsules represent well-recognized ferroptosis inhibitors, red capsules represent agents studied in cerebral I/R injury, purple for myocardial, yellow for lung, blue for renal and gray for intestinal. I/R, ischemia-reperfusion; GPX4, glutathione peroxidase 4; ACSL4, acyl-CoA synthetase long-chain family member 4; NCOA4, nuclear receptor coactivator 4; SLC7A11, solute carrier family 7 member 11; Nrf2, nuclear factor erythroid 2-related factor 2; HO-1, heme oxygenase 1; MAO-B, monoamine oxidase b; IREB2, iron response element-binding protein 2; TfR1, transferrin receptor 1; AMPKα2, adenosine 5′-monophosphate-activated protein kinase α2; p53, protein 53; FPN, ferroportin; USP7, ubiquitin-specific peptidase 7; USP14, ubiquitin-specific peptidase 14; USP19, ubiquitin-specific peptidase 19; USP22, ubiquitin-specific peptidase 22; TRF, transferrin; Gln, glutamine; MiR, microRNA; OxPCs, oxidized phosphatidylcholines; LncRNA, long non-coding RNA; ELAVL1, embryonic lethal-abnormal vision like protein 1; DNMT-1, DNA (cytosine-5)-methyltransferase 1; Nrf2, nuclear factor erythroid 2-related factor 2; TERT, telomerase reverse transcriptase; STAT3, signal transducer and activator of transcription 3; Sp1, special protein 1; TRPV1, transient receptor potential cation channel subfamily V member 1; LSD1, lysine- specific demethylase 1; Panx1, pannexin 1; CIRBP, cold-inducible RNA-binding protein; IDO, indoleamine 2,3-dioxy-genase 1; ALR, augmenter of liver regeneration; HUWE1, HECT domain-containing ubiquitin E3 ligase; SAT1, spermidine/spermine N1-acetyltransferase 1; PVT1, plasmacytoma variant 1; UBIAD1, UbiA prenyltransferase domain containing 1; PGE2, prostaglandin E2; Fer-1, ferrostatin-1; Lip-1, liproxstatin-1; DFO, deferoxamine; DXZ, dexrazoxane; α-Toc, α-tocopherol; GAA, gossypol acetic acid; C3G, cyanidin-3-glucoside; XN, xanthohumol; HC, histochrome; PDA NPs, polydopamine nanoparticles; APG, apigenin-7-O-β-D-(-6′′-p-coumaroyl)-glucopyranoside; CAR, carvacrol; Se, selenium; Res, resveratrol; CAT, capsiate; CY, carthamin yellow; ROSI, rosiglitazone; KF, kaempferol; DEX, dexmedetomidine; QCT, quercetin; FA, ferulic acid; iASPP, inhibitor of apoptosis-stimulating protein of p53; Eto, etomidate; NAR, naringenin; Bri, britanin; PA, pachymic acid; IA, isoliquiritin apioside.

A growing body of evidence suggests that ferroptosis plays a pivotal or even dominant role in the pathogenesis of I/R injuries, and strategies targeting ferroptosis to ameliorate I/R injury have recently been demonstrated in various preclinical I/R models (Figure 2). Although limited to animal and cell levels, protection against I/R injury mediated by chemical inhibitors of ferroptosis or the ablation of ferroptosis regulatory genes strongly suggests that ferroptosis is a promising target for drug development to prevent I/R injury. However, according to the available evidence, the regulatory mechanisms of ferroptosis in I/R injury are not fully understood. Moreover, the pharmacological mechanism of the currently known agents that inhibit ferroptosis in I/R, as well as their toxicity, side effects, safe doses and other issues, remain to be further elucidated in future preclinical and clinical trials. In conclusion, it is necessary to fully elucidate the mechanism of ferroptosis-mediated I/R injury and to identify ferroptosis regulators that can be safely targeted to alleviate I/R injury to develop effective therapeutic strategies.

Intensive study of ferroptosis has increasingly become a focus of therapeutic and prognosis improvement in a variety of diseases. The roles of ferroptosis in the pathogenesis of diseases and ferroptosis inhibition approaches are being extensively studied in multiple disease models, including but not limited to cancer [177], neurological diseases [178], infection and inflammation [179], etc., suggesting that targeting ferroptosis has great potential to improve therapeutic strategies for related systemic diseases. However, ferroptosis is still in the early stage of what should become a riveting research field, and the mechanism remains to be further examined, especially in specific disease models.

## Figures and Tables

**Table 1:** Therapeutic targets of ferroptosis in ischemia-reperfusion injury.

Therapeutic Targets	Diseases	Model	Key Mechanism	References
Tau	Cerebral IRI	In vivo	Tau-iron interaction, inhibit iron overload	[12]
Ferritin	Cerebral IRI	In vivo/In vitro	Regulate p53 and SLC7A11	[13,14]
Mitochondrial ferritin	Cerebral IRI	In vivo	Inhibit iron overload, inhibit lipid peroxidation	[15]
NCOA4 and USP14	Cerebral IRI	In vivo/In vitro	Promote ferritinophagy	[14]
UBIAD1	Cerebral IRI	In vivo/In vitro	Inhibit lipid peroxidation	[16]
PGE2	Cerebral IRI	In vivo/Human samples	Inhibit iron accumulation and lipid peroxidation	[17]
SAT1	Cerebral IRI	In vivo/In vitro	Transcriptional target of p53, induce lipid peroxidation	[18]
Thrombin	Cerebral IRI	In vivo/In vitro	Instigate esterification of ACSL4	[19]
LncRNA PVT1/miR-214	Cerebral IRI	In vivo/In vitro	Inhibit TfR1 and p53	[20]
Transferrin and glutamine	Myocardial IRI	In vivo/In vitro	Ferroptosis inducer	[21]
USP22	Myocardial IRI	In vivo/In vitro	Regulate SIRT1/p53/SLC7A11 axis	[22]
USP7	Myocardial IRI	In vivo/In vitro	Upregulate p53/TfR1 pathway	[23]
FPN	Myocardial IRI	In vivo/In vitro	Regulate iron homeostasis	[24]
DNMT-1	Myocardial IRI	In vivo/In vitro	Promote NCOA4-mediated ferritinophagy	[25]
OxPCs	Myocardial IRI	In vivo/In vitro	Suppress GPX4 activity	[26]
ELAVL1	Myocardial IRI	In vivo/In vitro	Promote autophagic ferroptosis	[27]
MiR-135b-3p	Myocardial IRI	In vivo/In vitro	Downregulate GPX4 expression	[28]
LncAABR07025387.1	Myocardial IRI	In vivo/In vitro	Sponge miR-205 to enhance ACSL4 expression	[29]
LncRNA Mir9-3hg	Myocardial IRI	In vivo/In vitro	Regulate Pum2/PRDX6 axis	[30]
MET	Liver IRI	In vivo/In vitro	Disrupt iron metabolism	[31]
HUWE1	Liver IRI	In vivo/In vitro/Human samples	Target TfR1 for proteasomal degradation	[32]
MiR-29a-3p	Liver IRI	In vivo/In vitro	Downregulate IREB2 expression	[33]
ALR	Renal IRI	In vitro	Anti-oxidant, upregulate GPX4 expression	[34]
Panx1	Renal IRI	In vivo/In vitro	Regulate HO-1, NCOA4 and FTH1	[35]
CIRBP	Renal IRI	In vivo/In vitro	Regulate ELAVL1 to promote ferritinophagy	[36]
Legumain	Renal IRI	In vivo/In vitro	Promote degradation of GPX4	[37]
IDO	Renal IRI	In vitro	Induce AhR-mediated ferroptosis	[38]
LSD1	Renal IRI	In vivo/In vitro	Upregulate TLR4/NOX4 pathway	[39]
MiR-182-5p and miR-378-3p	Renal IRI	In vivo/In vitro	Downregulate GPX4 and SLC7A11 expression	[40]
MiR-3587	Renal IRI	In vitro	Downregulate HO-1 expression	[41]
Sp1	Intestinal IRI	In vivo/In vitro/Human samples	Increase ACSL4 transcription	[42]
TRPV1	Intestinal IRI	In vivo/In vitro/Human samples	Upregulate GPX4 expression	[43]
Nrf2	IIR-induced lung injury	In vivo/In vitro	Upregulate SLC7A11-related axis	[44,45,46]
p53	IIR-induced lung injury	In vivo/In vitro	Regulate Nrf2 signaling pathway	[47]

Abbreviations: IRI, ischemia-reperfusion injury; p53, protein 53; SLC7A11, solute carrier family 7 member 11; NCOA4, nuclear receptor coactivator 4; USP14, ubiquitin-specific peptidase 14; UBIAD1, UbiA prenyltransferase domain containing 1; PGE2, prostaglandin E2; SAT1, spermidine/spermine N1-acetyltransferase 1; ACSL4, acyl-CoA synthetase long-chain family member 4; LncRNA, long non-coding RNA; PVT1, plasmacytoma variant 1; MiR, microRNA; TfR1, transferrin receptor 1; USP22, ubiquitin-specific peptidase 22; SIRT1, sirtuin-1; USP7, ubiquitin-specific peptidase 7; FPN, ferroportin; DNMT-1, DNA (cytosine-5)-methyltransferase 1; OxPCs, oxidized phosphatidylcholines; GPX4, glutathione peroxidase 4; ELAVL1, embryonic lethal-abnormal vision like protein 1; Pum2, pumilio RNA binding family member 2; PRDX6, peroxiredoxin 6; MET, macrophage extracellular trap; HUWE1, HECT domain-containing ubiquitin E3 ligase; IREB2, iron response element-binding protein 2; ALR, augmenter of liver regeneration; Panx1, pannexin 1; HO-1, heme oxygenase 1; FTH1, ferritin heavy chain 1; CIRBP, cold-inducible RNA-binding protein; IDO, indoleamine 2,3-dioxy-genase 1; AhR, aryl-hydrocarbon receptor; LSD1, lysine- specific demethylase 1; TLR4, toll like receptor 4; NOX4, NADPH oxidase 4; Sp1, special protein 1; TRPV1, transient receptor potential cation channel subfamily V member 1; Nrf2, nuclear factor erythroid 2-related factor 2; IIR, intestinal ischemia-reperfusion.

**Table 2:** Pharmacological therapeutic strategies targeting ferroptosis in ischemia-reperfusion injury.

Reagents	Diseases	Model	Function	References
Selenium compounds	Cerebral IRI	In vivo/In vitro	Drive GPX4 expression	[48,49,50]
Carvacrol	Cerebral IRI	In vitro	Upregulate GPX4 expression	[51]
Rehmannioside A	Cerebral IRI	In vivo/In vitro/Human samples	Activate SLC7A11/GPX4 axis	[52]
Galangin	Cerebral IRI	In vivo/In vitro	Activate SLC7A11/GPX4 axis	[53]
Carthamin yellow	Cerebral IRI	In vivo	Inhibit ACSL4 expression	[54]
Kaempferol	Cerebral IRI	In vitro	Activate Nrf2/SLC7A11/GPX4 axis	[55]
Liproxstatin-1	Cerebral, myocardial, lung, liver, intestinal IRI	In vivo/In vitro/Human samples	Inhibit lipid peroxidation	[12,42,56,57,58]
Ferrostatin-1	Cerebral, myocardial, lung, liver, renal IRI	In vivo/In vitro/Human samples	Inhibit lipid peroxidation	[12,20,26,31,59,60,61,62]
Deferoxamine	Myocardial, liver, renal IRI	In vivo/In vitro	Iron chelator	[21,31,61,62,63]
Dexrazoxane	Myocardial IRI	In vivo/In vitro	Iron chelator	[64]
Gossypol acetic acid	Myocardial IRI	In vivo/In vitro	Anti-oxidant/iron-chelating	[65]
Histochrome	Myocardial IRI	In vivo/In vitro	Anti-oxidant/iron-chelating	[66]
Cyanidin-3-glucoside	Myocardial IRI	In vivo/In vitro	Anti-oxidant	[67]
Xanthohumol	Myocardial IRI	In vivo/In vitro	Anti-oxidant/upregulate GPX4 expression	[68]
Etomidate	Myocardial IRI	In vivo	Upregulate Nrf2/HO-1 pathway	[69]
Naringenin	Myocardial IRI	In vivo/In vitro	Upregulate Nrf2/SLC7A11/GPX4 axis	[70]
Britanin	Myocardial IRI	In vivo/In vitro	Upregulate Nrf2/GPX4 axis	[71]
Propofol	Myocardial IRI	In vivo/In vitro	Regulate AKT/p53 pathway	[72]
Ferulic acid	Myocardial IRI	In vivo	Upregulate AMPKα2 expression	[73]
PDA NPs	Myocardial IRI	In vivo/In vitro	Inhibit iron deposition and lipid peroxidation	[74]
UAMC-3203	Myocardial IRI	In vivo	Inhibit lipid peroxidation	[75]
Resveratrol	Myocardial IRI	In vivo/In vitro	Regulate USP19-Beclin 1 autophagy/upregulate GPX4	[76]
Dexmedetomidine	Myocardial IRI	In vivo	Upregulate SLC7A11/GPX4 axis	[77]
Irisin	Lung, renal IRI	In vivo/In vitro	Upregulate Nrf2/HO-1 axis/upregulate GPX4	[60,78]
Rosiglitazone	Lung, intestinal IRI	In vivo/In vitro	Inhibit ACSL4 expression	[42,57]
α-tocopherol	Liver IRI	In vivo	Inhibit lipid peroxidation	[61]
Pachymic acid	Renal IRI	In vivo	Upregulate Nrf2 signaling pathway	[79]
16–86	Renal IRI	In vivo/In vitro	Inhibit lipid peroxidation	[80]
XJB-5-131	Renal IRI	In vivo	Inhibit lipid peroxidation/anti-oxidant	[81]
Quercetin	Renal IRI	In vivo/In vitro	Inhibit ATF3/SLC7A11/GPX4 axis	[82]
Nec-1f	Renal IRI	In vivo/In vitro	Inhibit RIPK1 kinase activity and ferroptosis	[83]
Entacapone	Renal IRI	In vivo/In vitro	Upregulate SLC7A11 repression	[84]
APG	Intestinal IRI	In vivo/In vitro	Inhibit MAO-B/anti-oxidant	[85]
Capsiate	Intestinal IRI	In vivo/In vitro	Enhance GPX4 expression/activate TRPV1	[43]
iASPP	IIR-induced lung injury	In vivo/In vitro	Upregulate Nrf2 signaling/p53 inhibitor	[47]
Isoliquiritin apioside	IIR-induced lung injury	In vivo/In vitro	Downregulate Hif-1α expression	[86]

Abbreviations: IRI, ischemia-reperfusion injury; GPX4, glutathione peroxidase 4; SLC7A11, solute carrier family 7 member 11; ACSL4, acyl-CoA synthetase long-chain family member 4; Nrf2, nuclear factor erythroid 2-related factor 2; HO-1, heme oxygenase 1; AKT, protein kinase B; p53, protein 53; AMPKα2, adenosine 5′-monophosphate-activated protein kinase α2; PDA NPs, polydopamine nanoparticles; USP19, ubiquitin-specific peptidase 19; ATF3, activation transcription factor 3; RIPK1, receptor interacting protein kinase 1; APG, apigenin-7-O-β-D-(-6′′-p-coumaroyl)-glucopyranoside; MAO-B, monoamine oxidase b; TRPV1, transient receptor potential cation channel subfamily V member 1; iASPP, inhibitor of apoptosis-stimulating protein of p53; IIR, intestinal ischemia-reperfusion.

## Abbreviations

ACSL4, acyl-CoA synthetase long-chain family member 4; AhR, aryl-hydrocarbon receptor; AKI, acute kidney injury; ALOX15, arachidonate 15-lipoxygenase; ALR, augmenter of liver regeneration; AMI, acute myocardial infarction; AMPK, adenosine monophosphate activated protein kinase; APG, apigenin-7-O-β-D-(-6′’-p-coumaroyl)-glucopyranoside; BMSC, bone marrow mesenchymal stem cell; Bri, britanin; CAR, carvacrol; CAT, capsiate; ceRNAs, competing endogenous RNAs; CIRBP, cold-inducible RNA-binding protein; COMT, catechol-O-methyltransferase; CoQ10, coenzyme Q10; COX-2, cyclooxygenase 2; C3G, cyanidin-3-glucoside; DEX, dexmedetomidine; DFO, deferoxamine; DM, diabetic myocardial; DNMT-1, DNA (cytosine-5)-methyltransferase 1; DHODH, dihydroorotate dehydrogenase; DXZ, dexrazoxane; ELAVL1, embryonic lethal-abnormal vision like protein 1; Eto, etomidate; FA, ferulic acid; Fer-1, ferrostatin-1; FOXC1, forkhead Box C1; FPN1, ferroportin 1; FSP1, ferroptosis suppressor protein 1; FtMt, mitochondrial ferritin; GAA, gossypol acetic acid; GCH1, GTP cyclohydrolase 1; GPX4, glutathione peroxidase 4; GSH, glutathione; GSH-GPX, glutathione-glutathione peroxidase; GSK3β, glycogen synthase kinase 3β; HC, histochrome; HK2, human kidney 2; HO-1, heme oxygenase 1; HUWE1, HECT domain-containing ubiquitin E3 ligase; IA, isoliquiritin apioside; iASPP, inhibitor of apoptosis-stimulating protein of p53; IDO, indoleamine 2,3-dioxygenase 1; IIR-ALI, intestinal ischemia-reperfusion induced acute lung injury; IIR, intestinal ischemia-reperfusion; I/R, ischemia-reperfusion; IRI, ischemia-reperfusion injury; IREB2, iron response element-binding protein 2; LIP, labile iron pool; Lip-1, liproxstatin-1; LIRI, lung I/R injury; LncRNA, long noncoding RNA; LOXs, lipoxygenases; LSD1, lysine-specific demethylase 1; MCAO, middle cerebral artery occlusion; MDA, malondialdehyde; MET, macrophage extracellular trap; MIRI, myocardial I/R injury; miRNA, microRNA; MMP, mitochondrial membrane potential; NAR, naringenin; NCOA4, nuclear receptor coactivator 4; Nrf2, nuclear factor erythroid 2-related factor 2; OxPCs, oxidized phosphatidylcholines; PA, pachymic acid; Panx1, pannexin 1; PDA NPs, polydopamine nanoparticles; PGE2, prostaglandin E2; PRDX6, peroxiredoxin 6; Pum2, pumilio RNA binding family member 2; PUFAs, polyunsaturated fatty acyls; PVT1, plasmacytoma variant 1; RCD, regulated cell death; Res, resveratrol; ROS, reactive oxygen species; ROSI, rosiglitazone; RPTECs, renal proximal tubular epithelial cells; SAT1, spermidine/spermine N1-acetyltransferase 1; Se, selenium; SIRT1, sirtuin-1; SLC7A11, solute carrier family 7 member 11; Sp1, special protein 1; STAT3, signal transducer and activator of transcription 3; TERT, telomerase reverse transcriptase; TFAP2c, transcription factor AP-2 gamma; TfR1, transferrin receptor 1; TRPV1, transient receptor potential cation channel subfamily V member 1; UBIAD1, ubiA prenyltransferase domain-containing 1; USP7, ubiquitin-specific protease 7; USP14, ubiquitin specific peptidase 14; USP19, ubiquitin-specific peptidase 19; USP22, ubiquitin-specific protease 22; XN, xanthohumol.
